# Effect of an intensive lifestyle intervention on the prevalence of metabolic syndrome and its components among overweight and obese adults

**DOI:** 10.1093/pubmed/fdz170

**Published:** 2019-12-16

**Authors:** M Guzmán, E Zbella, S Shah Alvarez, J L Nguyen, E Imperial, F J Troncale, C Holub, A K Mallhi, S VanWyk

**Affiliations:** 1 Division of Research & Development, Department of Medical Affairs, Medi-Weightloss, 509 South Hyde Park Avenue, Tampa, FL 33606, USA; 2 Florida Fertility Institute, 2454 N. McMullen Booth Road Suite 601, Clearwater, FL 33759, USA; 3 Department of Medical Affairs, Medi-Weightloss, 509 South Hyde Park Avenue, Tampa, FL 33606, USA; 4 Department of Pharmacy Practice, College of Pharmacy, Mercer University, 3001 Mercer University Drive, Atlanta, GA 30341, USA; 5 Iredell Primary Care for Women, 114 Gateway Blvd, Suite B, Mooresville, NC 28117, USA; 6 Section of Gastroenterology, Yale University School of Medicine, 333 Cedar St, New Haven, CT 06510, USA; 7 Department of Public Health, College of Education, Health and Human Services, California State University, 333 S. Twin Oaks Valley Road, San Marcos, CA 92096, USA; 8 Independent Consultant, 2518 69th Ave S, St. Petersburg, FL 33712, USA

**Keywords:** obesity, chronic disease

## Abstract

**Background:**

Despite the fact that up to a third of the global population has metabolic syndrome (MetS), it has been overlooked in clinical settings. This study assesses the impact of a physician-supervised nonsurgical weight management program on the prevalence of MetS and its key indicators.

**Methods:**

Four-hundred seventy-nine overweight and obese participants aged 19 years or older were included in a prospective longitudinal study. Changes in MetS and its key indicators were assessed using the binomial exact, chi-square and Wilcoxon signed-rank tests in an intent-to-treat study population. Differences in age strata were assessed using a generalized linear model.

**Results:**

Fifty-two percent of participants (*n* = 249) had MetS at baseline. Prevalence of MetS decreased steadily with significant changes from baseline observed at weeks 13 (31.8%, *P* < 0.0001), 26 (28.7%, *P* < 0.0012) and 39 (21.6%, *P* < 0.0002); changes from baseline were observed at week 52 as statistically significant (16.7%, *P* < 0.0012). Improvements in anthropometrics and levels of key indicators of MetS were observed throughout the study.

**Conclusion:**

These findings confirm that weight loss is inversely associated with prevalence of MetS and its key indicators among overweight and obese individuals. Future studies may benefit from a larger sample size and better retention (ClinicalTrials.gov ID: NCT03588117).

## Introduction

Metabolic syndrome (MetS) is a well-documented global health problem but often overlooked in clinical settings. Nearly 20–30% of the population in most countries are estimated to have MetS,^1^ highlighting a dire need for practitioners to understand what it is and what it means for their patients. MetS is recognized as a cluster of health indicators that can manifest as chronic conditions such as cardiovascular disease and type 2 diabetes.^2^ Today’s obesogenic environment and modern lifestyle contribute to unhealthy behaviors, such as overeating and physical inactivity, which increase the prevalence of chronic diseases.

A National Cholesterol Education Program-Adult Treatment Panel III (NCEP ATP III) report defined MetS as having three or more of the following: elevated waist circumference (WC), elevated triglycerides, reduced high-density lipoprotein (HDL) cholesterol, elevated blood pressure (BP) and elevated fasting glucose.^2^ These comorbidities are generally linked and commonly associated with increased adiposity, poor dietary habits and sedentary behavior, which are common characteristics of an unhealthy lifestyle.^3–5^

MetS is increasing in prevalence alongside the prevalence of obesity,^6^ an important determinant of MetS.^7^ Relative to normal and overweight populations, higher prevalence of MetS has been reported in obese populations, ranging from 59.6 to 75.7% of individuals studied.^7^^,^^8^^,^^9^ The Centers for Disease Control and Prevention found the prevalence of MetS among all adults in the United States increased from 25.3% in 1988–1994 to 34.2% in 2007–2012.^10^ Implementation of strategies for the prevention and treatment of MetS can have a substantial impact on reducing these trends.

Researchers emphasize the importance of lifestyle change as a component of MetS clinical management.^2^^,^^11^ Physician-supervised weight loss programs that include intensive lifestyle behavioral therapy have been shown to be an effective intervention to lose weight and improve MetS components ^7^^,^^12^^,^^13^; however, evidence on the efficacy of these programs for the treatment of MetS is limited and generally confined to secondary analyses.^14^ In the present study, we examine the impact of a physician-supervised non-surgical weight management program on prevalence of MetS. We hypothesize that the intervention will have a positive effect on the prevalence of MetS and its key indicators.

## Materials and methods

### Study locations and participants

**Fig. 1:**
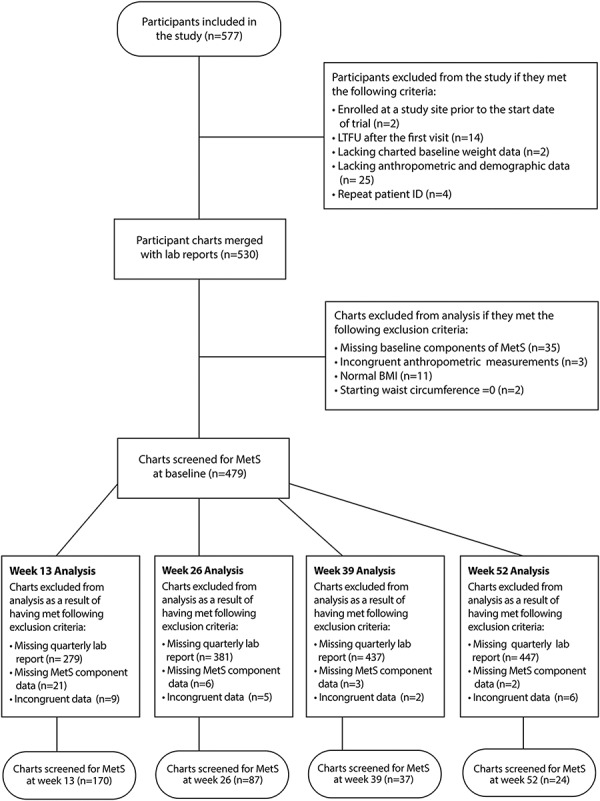
Flow of participants through the study.

A long-term prospective longitudinal study was conducted at five weight management clinic sites across the United States. Potential study participants were self-selected for intervention, having sought weight management assistance at one of the clinic sites and identified on their first clinic visit. They were enrolled in the study if the following inclusion criteria were met: (i) adult (age ≥ 18 years); (ii) overweight or obese (body mass index [BMI] ≥25 kg/m^2^); (iii) started the program on or after 1 March 2015; and (iv) provided informed consent. Consented study participants were dropped from data analysis if the following exclusion criteria were met: (i) previously enrolled at a clinic, (ii) lost to follow-up (LTFU) after the first visit, (iii) missing charted baseline weight data, (iv) missing anthropometric or demographic data, (v) missing follow-up lab reports; or (vi) found to be the subject of erroneous reporting (see Fig. 1 for further clarification). Of the 577 participants enrolled in the study, 479 met the inclusion and exclusion criteria (Fig. 1). Enrollment in the study was started on 1 March 2015 and ended on 31 July 2016. Participants were followed until the trial end date on 30 June 2017 or until LTFU. This study was conducted in consideration of the Declaration of Helsinki and Good Clinical Practice and registered with ClinicalTrials.gov (ID: NCT03588117).

### Intervention

Participants were enrolled in a physician-supervised non-surgical weight management program that provided weekly in-person counseling sessions with licensed clinicians. These in-person sessions were focused on educating participants on strategies to manage their weight and adopt a healthy lifestyle, such as a physical activity curriculum, self-monitoring through journaling, meal planning, portion control, and calorie restriction. Prescribed diets were individualized based on each participant’s behavior, level of physical activity and total energy expenditure (TEE). Nutraceutical supplements (e.g., *Hoodia gordonii* stem or Capsimax®), prescription appetite suppressants (i.e., phentermine or phendimetrazine), compounded injections (i.e., B vitamins and amino acids) and body composition analysis were additional components of the intensive lifestyle intervention. The program was divided into three phases: acute, short-term maintenance, and wellness.

The acute phase was a period of rapid weight loss that continued until achievement of a mutually determined goal weight between the participant and his/her clinician. Participants were prescribed an appetite suppressant if they met the following criteria: BMI ≥ 30 kg/m^2^ or BMI ≥ 27 kg/m^2^ with a comorbidity or adiposity of >25% in men and > 32% in women.^15^ After acute weight loss, participants entered the short-term maintenance phase. During this phase, participants incrementally increased their calorie intake to a well-balanced diet and discontinued the prescribed appetite suppressant, if applicable. Participants who achieved their goal weight or had completed a prescribed number of weeks in the program were permitted to enter the wellness phase. The primary goal of the wellness phase was to maintain weight loss by consuming up to a prescribed calorie load that was determined based on each participant’s TEE. This phase included monthly monitoring sessions. Each phase was undertaken with the supervision of a physician to ensure participants’ well-being and safety. Throughout the program, participants were encouraged to engage in 150–250 minutes of exercise weekly, performing cardiovascular, resistance and flexibility training.

### Data collection

Every participant completed a baseline survey during their first visit. A physical or electronic (delivered via tablet) copy of the survey was provided to the participants. Electronic copies of the surveys were provided through electronic data capture systems i.e. Clinical Studio and SurveyGizmo (Crucial Data Solutions, Inc., Reno, Nevada; SurveyGizmo: Boulder, Colorado). The baseline survey included questions on demographics, medical history, behavior and current medications. Quarterly surveys were completed at weeks 13, 26, 39 and 52 and included questions on medication. Anthropometric data and any adverse events were recorded in the electronic health record by the clinician. All participants received a complete medical exam during their first visit, including medical history, physical examination, vital sign measurements, electrocardiogram, urinalysis, lipid panel, comprehensive metabolic panel and hemoglobin A1c (HbA1c test). A lipid panel, comprehensive metabolic panel and HbA1c test were also conducted at each quarterly visit. Throughout the program, weight and body composition were recorded using a Tanita Scale**®** (Tanita Corporation of America, Inc., Arlington Heights, Illinois). BP was measured once at baseline and at each subsequent visit after 5 minutes of rest using an appropriately sized BP cuff (3 M™ Littmann® manual or Omron® automatic). WC was measured at the narrowest point of hip area or the midpoint between the lower costal (10th rib) border and the iliac crest (upper hip bone). During the wellness phase, a ReeVue™ Indirect Calorimeter (Korr™ Medical Technologies Inc., Salt Lake City, Utah) was used to calculate TEE.

### MetS screening criteria

Anthropometrics and lab results recorded at baseline and each quarterly visit were used to identify patients who met the criteria for MetS. Using the NCEP ATP III criteria,^1^ MetS was determined to be present if three or more of the following five criteria were met: (i) elevated WC: > 102 cm in men or > 88 cm in women; (ii) elevated triglycerides: ≥1.7 mmol/l or on drug treatment for elevated triglycerides; (iii) reduced HDL-C: < 1.03 mmol/l in men or 1.29 mmol/l in women or on drug treatment for reduced HDL-C; (iv) elevated BP: ≥130 mm Hg systolic BP (SBP) or ≥ 85 mm Hg diastolic BP (DBP) or on antihypertensive drug treatment in patients with a medical history of hypertension; and (v) elevated fasting plasma glucose (FPG): ≥5.5 mmol/l or on drug treatment for elevated glucose. HbA1c ≥5.7% was used as a surrogate measure of elevated FPG for qualification of MetS,^16^ such that participants with FPG < 5.5 mmol/L and/or HbA1c > 5.7% were determined to have met the criterion.

### Statistical analysis

Changes in weight, BP and WC were examined using a modified intent-to-treat (ITT) sample with the last observation carried forward (LOCF) method to impute missing values. The modified ITT sample was defined as all participants who enrolled in the study with at least one post-baseline measurement of weight. In an effort to maximize viability, participants’ laboratory data were accepted and stratified in ranges that extended to 3 weeks before and after each target appointment week such that the week 13, 26, 39 and 52 assessments could include data from week ranges 10–16, 23–29, 36–42 and 49–55, respectively. However, the data collected outside of the designated weekly ranges were not included in the analysis. This method may have reduced the sample size at subsequent follow-ups. Reduction in sample size was not expected to reflect true attrition in a traditional sense.

For baseline demographics (Table 1), between-group comparisons of categorical and continuous variables were performed using χ^2^ tests or analysis of variance. For weeks 13, 26, 39 and 52, weight, BMI, body composition and components of MetS were explored for normality using the Shapiro-Wilk test of normality, and differences in mean values were assessed using the Wilcoxon signed-rank test. The prevalence rates of MetS and its positive health indicators were assessed with the binomial exact test and χ^2^ test at weeks 13, 26, 39 and 52, relative to baseline. MetS was defined as a summation of MetS positive health indicator screenings.

**Table 1:** Baseline demographics and characteristics

Variables	MetS (*n* = 249)^b^		Non-MetS (*n* = 230)^c^		*P*-value^a^
Age (years)					
Mean (SD)	48.6 (12.2)		43.3 (10.8)		<0.0001
Starting BMI (kg m^−2^)					
Mean (SD)	37.5 (6.8)		33.0 (5.6)		<0.0001
	*n*	%	*n*	%	
Age groups					
≤50 years old	133	53.4	171	74.3	<0.0001
>50 years old	116	46.6	59	25.7	
Sex					
Female	208	83.5	212	92.2	0.004
Male	41	16.5	18	7.8	
Obesity categories					
Overweight	23	9.3	78	34.0	<0.0001
Obese I	73	29.3	93	40.4	
Obese II	83	33.3	31	13.5	
Obese III	70	28.1	28	12.1	
Race					
White	186	81.6	195	90.3	0.0087
Non-White	42	18.4	21	9.7	
Education					
Advanced degree	51	20.7	49	21.4	0.0269
Bachelor’s degree	68	27.5	83	36.2	
Associate’s degree	35	14.2	39	17.0	
Less than Associate degree	93	37.6	58	25.3	
Annual household income					
$100,000 or above	95	40.2	111	50.6	0.0665
$50,000–$99,999	95	40.3	77	35.2	
Under $49,999	46	19.5	31	14.2	
Appetite suppressant					
Prescription appetite suppressant	157	63.1	152	66.1	0.7098
Herbal dietary supplement	24	9.6	18	7.8	
No appetite suppressant	68	27.3	60	26.1	
	**Mean**	**(SD)**	**Mean**	**(SD)**	
Body composition					
Weight (kg)	104.11	(20.96)	90.80	(17.97)	<0.0001
FM (kg)	47.75	(13.14)	38.84	(12.03)	<0.0001
Fat-free mass (kg)	56.27	(11.08)	51.90	(9.07)	<0.0001
Body fat %	45.52	(5.58)	42.21	(5.90)	<0.0001
WC (cm)					
Female	108.68	(13.48)	98.24	(13.76)	<0.0001
Male	122.69	(10.13)	110.49	(14.38)	0.0004
Serum glucose (mmol/l)	5.47	(1.65)	4.66	(0.68)	<0.0001
BP (mm Hg)					
SBP	128.28	(13.14)	120.03	(12.65)	<0.0001
DBP	81.64	(7.08)	77.70	(7.19)	<0.0001
					
Triglycerides (mmol/l)	2.02	(1.15)	1.12	(0.48)	<0.0001
HDL (mmol/l)					
Female	1.32	(0.51)	1.64	(0.35)	<0.0001
Male	1.11	(0.28)	1.27	(0.26)	0.0409

SD, standard deviation.

^a^ *P*-values compare groups by χ^2^ tests for categorical variables (proportions) and analysis of variance for continuous variables (means).

^b^For race: *N* = 228, education: *N* = 247, annual household income: *N* = 236, FM: *N* = 248, fat-free mass: *N* = 248, body fat %: *N* = 248, serum glucose: *N* = 248.

^c^For race: *N* = 216, education: *N* = 229, annual household income: *N* = 219, serum glucose: *N* = 228.

A generalized linear model was created to assess the least square mean change and standard error in two age groups adjusted for covariates that included sex, starting BMI, race, education, annual household income and use of prescription appetite suppressants. In all analyses, a *P*-value of < 0.05 was considered statistically significant. All statistical analyses were performed using SAS 9.4 (SAS Institute Inc., Cary, North Carolina).

## Results

### Baseline prevalence of MetS and its individual components

The final sample was comprised of 479 participants of which 249 (52%) were found to have met the criteria for MetS at baseline. Upon entering the study, participants screened positive at varying rates for individual components of MetS, including lowered HDL-C and elevated WC, triglycerides, BP and FPG levels (Table 2). Participants were found to test positive at baseline for five (8.98%), four (15.66%), three (27.35%), two (24.43%), one (18.58%) or zero (5%) individual components of MetS. The final sample was stratified by MetS status such that the demographics of participant groups screened as MetS positive or negative were examined individually (Table 1).

**Table 2:** Prevalence of MetS and its individual components at baseline, week 13, week 26, week 39 and week 52

Variables	Week 13^a^					Week 26^a^				
	Baseline (*n* = 170)		Week 13 (*n* = 170)			Baseline (*n* = 87)		Week 26 (*n* = 87)		
	%	95% CI^b^	%	95% CI^b^	*P*-value^c^	%	95% CI^b^	%	95% CI^b^	*P*-value^c^
		(Lower CI–upper CI)		(Lower CI–upper CI)			(Lower CI–upper CI)		(Lower CI–upper CI)	
Prevalence of MetS (%)	**58.2**	(0.50–0.66)	**31.8**	(0.25–0.39)	<0.0001	**52.9**	(0.42–0.64)	**28.7**	(0.20–0.39)	0.0012
Prevalence of individual components of MetS (%)										
Elevated blood glucose	55.9	(0.48–0.63)	43.5	(0.36–0.51)	0.0227	50.6	(0.40–0.61)	40.2	(0.30–0.51)	0.1706
Elevated triglycerides	40.0	(0.33–0.48)	23.5	(0.17–0.31)	0.0011	33.3	(0.24–0.44)	20.7	(0.13–0.31)	0.0604
Large WC	86.5	(0.80–0.91)	51.8	(0.44–0.59)	<0.0001	88.5	(0.80–0.94)	40.2	(0.30–0.51)	<0.0001
Lowered HDL	38.2	(0.31–0.46)	31.8	(0.25–0.39)	0.2110	29.9	(0.21–0.41)	25.3	(0.17–0.36)	0.4975
High BP	54.7	(0.47–0.62)	40.6	(0.33–0.48)	0.0092	60.9	(0.50–0.71)	34.5	(0.25–0.45)	0.0005
	Week 39^a^					Week 52^a^				
	Baseline (*n* = 37)		Week 39 (*n* = 37)			Baseline (*n* = 24)		Week 52 (*n* = 24)		
	%	95% CI^b^	%	95% CI^b^	*P*-value^c^	%	95% CI^b^	%	95% CI^b^	*P*-value^c^
		(Lower CI–upper CI)		(Lower CI–upper CI)		(Lower CI–upper CI)			(Lower CI–upper CI)	
										
Prevalence of MetS (%)	**64.9**	(0.47–0.80)	**21.6**	(0.10–0.38)	0.0002	**62.5**	(0.41–0.81)	**16.7**	(0.05–0.37)	0.0012
Prevalence of individual components of MetS (%)										
Elevated blood glucose	54.1	(0.37–0.71)	24.3	(0.12–0.41)	0.0088	50.0	(0.29–0.71)	25.0	(0.10–0.47)	0.0736
Elevated triglycerides	35.1	(0.20–0.53)	16.2	(0.06–0.32)	0.0625	25.0	(0.10–0.47)	16.7	(0.05–0.37)	0.4772
Large WC	89.2	(0.75–0.97)	40.5	(0.25–0.58)	<0.0001	83.3	(0.63–0.95)	33.3	(0.16–0.55)	0.0004
Lowered HDL	35.1	(0.20–0.53)	5.4	(0.01–0.18)	0.0015	37.5	(0.19–0.59)	8.3	(0.01–0.27)	0.0162
High BP	70.3	(0.53–0.84)	43.2	(0.27–0.61)	0.0190	75.0	(0.53–0.90)	45.8	(0.26–0.67)	0.0388

^a^Only participants with data from baseline and the follow-up week of interest (e.g., week 13, 26, 39 or 52) were included in the analysis.

^b^95% CIs for point prevalence at baseline and follow-up computed using the binomial exact test.

^c^ *P*-values computed using χ^2^ test for prevalence comparison at baseline versus follow-up weeks.

### Prevalence of MetS and its individual components

Participants experienced a significant reduction in the prevalence of MetS as they progressed in the study (Table 2). Prevalence of MetS among participants decreased with significant changes from baseline observed at weeks 13, 26, 39 and 52.

As the study progressed, participants screened positive for individual components of MetS at lower rates with significant changes from baseline observed throughout the study; however, changes in prevalence of reduced HDL-C at weeks 13 and 26 were nonsignificant but were significant at weeks 39 and 52. Elevated triglycerides at weeks 26, 39 and 52 were also not significant (Table 2). A relationship was observed between weight loss and levels of individual components of MetS, such that levels were largely found to continue to improve with greater weight loss at each subsequent follow-up (Table 3). Mean levels of individual components significantly improved throughout the study; however, changes in levels of HDL-C at week 13 and FPG at weeks 39 and 52 were not significant.

**Table 3:** Mean change in BMI, body composition and individual component of MetS at week 13, week 26, week 39 and week 52

Variables	Baseline vs. Week 13 (*n* = 170)^a^			Baseline vs. Week 26 (*n* = 87)^a^			Baseline vs. Week 39 (*n* = 37)^a^			Baseline vs. Week 52 (*n* = 24)^a^		
	Mean			Mean			Mean			Mean		
	Baseline ± SD	Change ± SD	*P*-value	Baseline ± SD	Change ± SD	*P*-value	Baseline ± SD	Change ± SD	*P*-value	Baseline ± SD	Change ± SD	*P*-value
BMI (Kg/m^−2^)	35.42 ± (6.47)	−4.71 ± (1.78)	<0.0001	35.54 ± (6.67)	−6.53 ± 3.14	<0.0001	36.72 ± (5.57)	−7.36 ± (4.46)	<0.0001	34.66 ± (7.08)	−6.35 ± (3.35)	<0.0001
Body composition												
Weight (kg)	98.20 ± (20.85)	−13.13 ± (5.45)	<0.0001	98.45 ± (21.94)	−18.31 ± (9.71)	<0.0001	104.38 ± (20.06)	−21.22 ± (13.77)	<0.0001	96.82 ± (20.18)	−17.70 ± (9.32)	<0.0001
FM (kg)	43.99 ± (12.61)	−10.11 ± (5.08)	<0.0001	44.34 ± (12.80)	−14.19 ± (8.29)	<0.0001	47.51 ± (11.46)	−16.78 ± (12.09)	<0.0001	43.05 ± (13.13)	−14.08 ± (8.16)	<0.0001
Fat-free mass (kg)	54.05 ± (10.67)	−2.97 ± (2.72)	<0.0001	53.78 ± (11.35)	−3.96 ± (3.23)	<0.0001	56.26 ± (12.00)	−4.05 ± (3.45)	<0.0001	52.50 ± (8.91)	−3.05 ± (3.26)	0.000
Body fat %	44.38 ± (5.28)	−5.14 ± (3.32)	<0.0001	44.74 ± (4.93)	−7.71 ± (4.92)	<0.0001	45.62 ± (5.47)	−8.70 ± (6.78)	<0.0001	44.34 ± (5.80)	−8.13 ± (5.38)	<0.0001
MetS Components												
WC (cm)												
Total	105.54 ± (15.12)	−13.88 ± (8.36)	<0.0001	106.52 ± (15.37)	−18.19 ± (7.96)	<0.0001	109.67 ± (14.49)	−21.62 ± (13.63)	<0.0001	105.52 ± (15.67)	−18.52 ± (7.75)	<0.0001
Female	103.10 ± (13.87)	−13.14 ± (5.97)	<0.0001	104.04 ± (14.33)	−17.12 ± (7.09)	<0.0001	106.21 ± (13.70)	−19.77 ± (13.88)	<0.0001	103.23 ± (15.41)	−17.78 ± (7.85)	<0.0001
Male	122.83 ± (12.27)	−19.17 ± (17.12)	<0.0001	125.60 ± (8.20)	−26.42 ± (9.80)	0.002	124.46 ± (6.09)	−29.57 ± (9.66)	0.016	121.50 ± (3.20)	−23.71 ± (5.29)	0.250
Serum glucose (mmol/l)	5.06 ± (1.46)	−0.21 ± (0.93)	0.007	5.05 ± (1.19)	−0.38 ± (0.95)	0.001	5.21 ± (1.23)	−0.38 ± (1.08)	0.072	4.88 ± (0.56)	−0.12 ± (0.78)	0.477
BP (mm Hg)												
SBP	125.35 ± (13.81)	−6.82 ± (14.36)	<0.0001	125.93 ± (13.49)	−10.32 ± (13.05)	<0.0001	125.78 ± (12.26)	−6.57 ± (11.35)	0.001	126.38 ± (15.55)	−7.54 ± (14.11)	0.006
DBP	79.93 ± (6.85)	−2.83 ± (8.40)	<0.0001	80.29 ± (6.59)	−4.47 ± (8.54)	<0.0001	82.32 ± (5.15)	−5.65 ± (6.88)	<0.0001	80.83 ± (7.89)	−4.63 ± (9.77)	0.017
												
Triglycerides (mmol/l)	1.62 ± (0.87)	−0.52 ± (0.81)	<0.0001	1.49 ± (0.68)	−0.48 ± (0.98)	<0.0001	1.47 ± (0.57)	−0.54 ± (0.55)	<0.0001	1.54 ± (1.29)	−0.76 ± (1.29)	<0.0001
HDL (mmol/l)												
Total	1.46 ± (0.58)	−0.01 ± (0.47)	0.389	1.49 ± (0.43)	0.18 ± (0.30)	<0.0001	1.47 ± (0.57)	0.25 ± (0.24)	<0.0001	1.46 ± (0.38)	0.35 ± (0.22)	<0.0001
Female	1.50 ± (0.60)	0.04 ± (0.49)	0.820	1.52 ± (0.43)	0.17 ± (0.28)	<0.0001	1.47 ± (0.43)	0.23 ± (0.23)	<0.0001	1.46 ± (0.37)	0.36 ± (0.23)	<0.0001
Male	1.19 ± (0.29)	0.16 ± (0.24)	0.002	1.27 ± (0.35)	0.28 ± (0.41)	0.074	1.33 ± (0.41)	0.35 ± (0.26)	0.031	1.47 ± (0.54)	0.24 ± (0.04)	0.250

SD, standard deviation.

^a^Only participants with data from baseline and the follow-up week of interest (e.g., week 13, 26, 39 or 52) were included in the analysis.

### Change in body composition

Improvements in measures of body composition were observed throughout the study. At baseline, participants meeting the criteria for MetS were noted as having less favorable measurements for weight, fat mass (FM) and BMI (Table 1). Over the course of participation in the study, the percentage of participants with overweight or obesity significantly decreased relative to baseline (weeks 13: 85.29%, 26: 75.86%, 39: 89.19%, 52: 66.67%; *P* < 0.0001 in all cases). Among all participants, mean levels of individual anthropometrics significantly decreased from baseline throughout the study such that clinically relevant changes in weight, FM and body fat percentage (BFP) were observed at weeks 13 (weight: −13.13 kg; FM: −10.11 kg; BFP: −5.14%), 26 (weight: −18.31 kg; FM: −14.19 kg; BFP: −7.71%), 39 (weight: −21.22 kg; FM: −16.78 kg; BFP: −8.70%) and 52 (weight: −17.70 kg; FM: −14.08 kg; BFP: −8.13%) (Table 3). As a percentage of starting body weight, participant weight loss was observed to improve at weeks 13 (13.3%; 95% confidence interval [CI]: 12.7–13.9), 26 (18.2%, CI: 16.7–19.6) and 39 (19.3%, CI: 16.1–22.5). Accordingly, participant BMI was also found to improve (Table 3). Relatively mild variations in all anthropometrics at week 52 may be explained by the lower starting weight of the cohort (i.e., starting weight of participants who were retained to week 52). Participant fat-free mass was also found to significantly decrease from baseline at each follow-up, but such changes were noted as being less severe overall.

### Individual components of MetS and age groups

A stratified comparison of least square mean change in individual components of MetS revealed no consistently significant differences between participant groups aged ≤50 years and > 50 years. Differences between age groups were examined at each follow-up period, adjusting for BMI, sex, race, education, income levels and use of appetite suppressants. Statistically significant differences in HDL-C at week 26, in DBP at week 39 and in SBP at week 52 were observed (Table 4); no statistically significant differences between participant groups were observed at week 13.

**Table 4:** Least square mean change in body composition and MetS components adjusted for starting BMI, sex, race, income, education and appetite suppressants

Variables	Week 13^a^					Week 26^a^				
	Age group 1 (age ≤ 50)		Age group 2 (age > 50)		*P*-value	Age group 1 (age ≤ 50)		Age group 2 (age > 50)		*P*-value
	Mean	SE	Mean	SE	GP1 vs.GP2	Mean	SE	Mean	SE	GP1 vs.GP2
Weight change (kg)	−13.66	(0.97)	−13.44	(0.97)	0.785	−24.70	(2.38)	−25.21	(2.43)	0.777
FM change (kg)	−10.15	(0.98)	−9.66	(0.97)	0.531	−20.56	(2.26)	−19.90	(2.29)	0.685
Fat-free mass change (kg)	−3.29	(0.59)	−3.55	(0.58)	0.582	−3.16	(0.93)	−4.35	(0.94)	0.077
Body fat % change	−4.84	(0.69)	−4.35	(0.69)	0.374	−11.70	(1.55)	−10.89	(1.57)	0.467
WC change (cm)	−13.55	(1.41)	−13.69	(1.40)	0.901	−25.34	(2.24)	−25.44	(2.29)	0.951
FPG change (mmol/l)	−0.20	(0.21)	−0.62	(0.21)	0.014	−0.87	(0.33)	−1.15	(0.33)	0.247
Triglycerides change (mmol/l)	−0.39	(0.16)	−0.60	(0.15)	0.098	−0.69	(0.34)	−1.14	(0.34)	0.078
HDL change (mmol/l)	0.08	(0.11)	0.18	(0.11)	0.316	0.13	(0.10)	0.32	(0.10)	0.014
SBP change (mm Hg)	−6.49	(2.97)	−14.46	(2.96)	0.001	−5.65	(4.76)	−11.14	(4.86)	0.128
DBP change (mm Hg)	−2.73	(1.99)	−3.75	(1.98)	0.527	−2.95	(3.23)	−2.76	(3.29)	0.935
	WEEK 39^a^					WEEK 52^a^				
	Age group 1 (age ≤ 50)		Age group 2 (age > 50)		*P*-value	Age group 1 (age ≤ 50)		Age group 2 (age > 50)		*P*-value
	Mean	SE	Mean	SE	GP1 vs.GP2	Mean	SE	Mean	SE	GP1 vs.GP2
Weight change (kg)	−23.75	(4.04)	−24.10	(3.87)	0.919	−22.34	(8.47)	−18.29	(7.95)	0.548
FM change (kg)	−19.88	(4.06)	−19.10	(3.74)	0.811	−14.33	(8.93)	−6.72	(8.94)	0.239
Fat-free mass change (kg)	−2.81	(1.50)	−4.09	(1.39)	0.293	−1.70	(2.97)	−4.35	(2.98)	0.219
Body fat % change	−11.62	(2.86)	−10.81	(2.64)	0.722	−8.00	(7.24)	−1.27	(7.25)	0.203
WC change (cm)	−27.02	(5.53)	−30.00	(5.30)	0.536	−26.82	(8.90)	−21.29	(8.35)	0.439
FPG change (mmol/l)	−0.82	(0.47)	−1.39	(0.45)	0.173	0.10	(0.58)	0.33	(0.56)	0.622
Triglycerides change (mmol/l)	−0.64	(0.24)	−0.80	(0.23)	0.430	−0.45	(0.27)	−0.11	(0.25)	0.134
HDL change (mmol/l)	0.28	(0.12)	0.28	(0.12)	0.966	0.23	(0.25)	0.10	(0.23)	0.507
SBP change (mm Hg)	−7.94	(5.45)	−4.56	(5.21)	0.476	−14.06	(12.57)	−34.31	(11.80)	0.068
DBP change (mm Hg)	−10.78	(2.56)	−4.14	(2.45)	0.007	0.92	(7.39)	−11.87	(6.93)	0.053

SE, standard error; GP1, age group 1 (< 50); GP2, age group 2 (>50).

^a^Only participants with data from baseline and the follow-up week of interest (e.g., week 13, 26, 39, or 52) were included in the analysis

## Discussion

### Main findings of this study

The present study investigated the prevalence of MetS and its individual components among individuals classified as overweight and obese. It demonstrated that a nonsurgical weight management program reduced the prevalence of MetS and improved the levels of key indicators of MetS among overweight or obese participants who were retained in the program for up to 52 weeks. Important differences between the study participants and other overweight or obese populations were additionally noted and expected to be attributable to the demographics of the participants.

### What is already known on this topic

Previous studies of overweight or obese populations reported higher overall prevalence of MetS (59.6–75.7%) than was observed in this study (52%).^7^^,^^8^^,^^9^ This difference may be explained by dissimilarities in socioeconomic status (SES) and education level. Previous research has shown an inverse association between prevalence of MetS and demographics, such as SES and education.^9^^,^^17^ Yet, it has been noted that there have been people of high SES with obesity who were also considered metabolically healthy, such that obesity is present with reduced or without the burden of any metabolic disorder.^18^ More than 50% of participants reported an annual household income of at least $100 000, and 45% reported completing at least a bachelor’s degree (Table 1). These percentages were higher than those observed in prior research that focused on people with obesity.^9^

### What this study adds

The study found a consistent decrease in the prevalence of MetS and body weight from baseline to week 52, indicating that the intervention alongside weight loss is strongly associated with prevalence of MetS. Prior research has indicated that obesity is a strong predictor of MetS and that weight loss may be the best treatment option.^19^ As 95% of this study’s participants were found to have at least one component of MetS at baseline, improvements in weight loss were expected to positively impact individual components of MetS.

Gradual improvements in the components of MetS were observed during the study period. At week 13, for instance, statistically significant improvements were observed in all components except HDL. As seen in most other components, however, the results show a statistically significant improvement in HDL levels at weeks 39 and 52, suggesting that a longer stay in the program and greater weight loss may be related to significant improvement in HDL levels. Previous studies have also demonstrated an increase in HDL levels with weight loss ^20^^,^^21^ and suggested that improvement in HDL may occur due to weight loss after body weight has been stable for some time.^21^^,^^22^ This improvement may be attributable to the increased activity of adipose tissue lipoprotein lipase after weight stabilization at a reduced body weight, although reduced lipase activity during the active weight loss period has also been reported.^23^^,^^24^ Therefore, this study augments the findings of previous weight loss research that investigated the impact of weight loss on cardiometabolic health indicators.

Improvements were observed for components other than HDL throughout the study. Significant reductions in the mean FPG occurred at weeks 13 (0.21 mmol/l) and 26 (0.38 mmol/l); however, the mean reduction failed to reach a level of statistical significance thereafter. Prior research has shown similar results and reported statistically significant glycemic improvements only during the early phase of a lifestyle intervention program.^25^ The relatively small change in participant FPG levels observed throughout this study may be reflective of the fact that the majority of participants already had normal levels at baseline (Table 1).

### Limitations of this study

The limitations of this study include the following: (1) the sample only included overweight or obese participants, and the results may not be generalized to other populations; (2) the age strata were small which may have reduced the statistical power to detect any significant differences between groups; (3) the study had a low retention rate over a period of 1 year; and (4) the LOCF method was used to handle missing data of WC and BP, which may have overestimated the prevalence of these components at each follow-up. However, this study has several strengths, including prospective design, repeated measures data, long-term follow-up period of 1 year and adjustment for social demographic factors.

In conclusion, MetS was prevalent in more than half of study participants, including overweight and obese individuals who enrolled in a physician-supervised nonsurgical weight management program. The program was found to contribute to improvements in health outcomes and reductions in the prevalence of MetS among participants throughout the study period. These findings appear to support the significance of weight loss as a crucial aspect of MetS clinical management. More emphasis should be placed on intensive lifestyle interventions to combat MetS in the overweight and obese populations.

## Notes

**M. Guzmán**, Chief Science Officer

**E. Zbella**, Medical Director

**S. Shah Alvarez**, Associate Medical Director

**J. L. Nguyen**, Assistant Professor

**E. Imperial**, Family Medicine Physician

**F. J. Troncale**, Assistant Clinical Professor of Medicine (emeritus)

**C. Holub**, Assistant Professor

**A. K. Mallhi**, Clinical Research Scientist

**S. VanWyk**, Independent Consultant.
